# Negative Pressure Wound Therapy With Saline Instillation Improves Early Healing in Purulent Soft-Tissue Disease

**DOI:** 10.7759/cureus.100098

**Published:** 2025-12-25

**Authors:** Maxim A Chinikov, Valery A Kislyakov, Malik K Al-Ariki, Mohareb A Antone Lamey, Liya K Misharina, Abubakar I. Sidik

**Affiliations:** 1 Department of Hospital Surgery; Pediatric Surgery, Medical Institute of the Peoples' Friendship University of Russia Named After Patrice Lumumba, Moscow, RUS; 2 Department of Cardiovascular Surgery, Medical Institute of the Peoples' Friendship University of Russia Named After Patrice Lumumba, Moscow, RUS

**Keywords:** granulation tissue formation, negative pressure wound therapy (npwt), negative pressure wound therapy with instillation and dwell time, purulent inflammation, surgical debridement, vacuum-assisted wound closure, wound planimetric analysis

## Abstract

Background

Negative pressure wound therapy (NPWT) is widely used in the treatment of purulent-inflammatory soft tissue diseases, while negative pressure wound therapy with instillation (NPWT-i) has recently emerged as a promising approach to enhance wound healing.

Methods

We conducted a prospective, single-center randomized study, including 29 patients with purulent-inflammatory soft tissue infections of the trunk and extremities treated between December 2024 and June 2025. Patients were randomized to conventional NPWT (Group I, n=17) or NPWT-i with normal saline (Group II, n=12) after accounting for exclusions during follow-up. All patients underwent standardized surgical debridement protocols, and outcomes were assessed by planimetric wound analysis, serum C-reactive protein (CRP) measurement, number of repeated surgical debridements, and length of hospital stay.

Results

By day 7 after initial surgical debridement, patients in the NPWT-i group demonstrated significantly greater granulation tissue formation (70.5 ± 4.6% vs. 48.7 ± 4.1%), reduced fibrin coverage (17.2 ± 1.4% vs. 28.1 ± 5.2%), and smaller areas of necrosis (12.3 ± 3.1% vs. 23.6 ± 2.8%) compared to the NPWT group (all p < 0.05). CRP levels decreased more rapidly with NPWT-i (49.4 ± 29.1 mg/L vs. 96.3 ± 14.2 mg/L, p < 0.05). Patients in the NPWT-i group required fewer repeat debridements (3.1 ± 0.7 vs. 5.4 ± 1.2, p < 0.05) and had a shorter mean hospital stay (32.5 ± 4.2 vs. 41.7 ± 6.1 days, p < 0.05).

Conclusion

NPWT-i accelerates wound healing, reduces inflammation, decreases the number of repeated surgical interventions, and shortens hospitalization compared with standard NPWT. This justifies NPWT-i consideration as an advanced therapeutic option, particularly for the management of extensive purulent wounds where efficient and early wound-bed preparation is critical. Further large-scale, multicenter trials are warranted to confirm these preliminary findings, evaluate long-term outcomes, and establish standardized instillation protocols.

## Introduction

Purulent-inflammatory diseases of the soft tissues of the trunk and extremities remain one of the most frequent reasons for hospitalization in surgical departments. The most common causative agents of these infections are Staphylococci, Streptococci, and anaerobic bacteria [1]. These conditions are typically characterized by a rapid progression of the inflammatory process with significant tissue destruction [2,3].

In the treatment of patients with purulent-inflammatory soft tissue diseases, the method of topical negative pressure wound therapy (NPWT) has been widely used for over 20 years [4]. However, one of the newer treatment modalities is negative pressure wound therapy with instillation (NPWT-i) [5].

Since the beginning of the twenty-first century, the possibility of combined treatment using topical negative pressure with wound irrigation has been explored [6]. An example is NPWT-i of solutions, which has become more convenient for clinical application following the introduction of devices with improved irrigation systems. This method is based on the concept of wound cleansing through the repeated introduction of a solution for a specified dwell time, followed by its aspiration [7].

Additional beneficial effects of using NPWT-i include: reduction of bacterial bioburden in tissues; maintenance and preservation of a moist wound environment; and the possibility of topical administration of medicinal agents [8].

There are limited reports in the literature on the positive effect of NPWT-i of normal saline for the treatment of purulent-inflammatory soft tissue diseases [9,10]. Therefore, this prospective, randomized study aimed to directly compare the clinical efficacy and early wound outcomes of NPWT-i using normal saline versus standard NPWT in patients with purulent-inflammatory soft tissue infections of the trunk and extremities.

## Materials and methods

The study protocol was reviewed and approved by the Academic Council of the Medical Institute of the Patrice Lumumba Russian University of Peoples' Friendship (RUDN University), Minutes No. 4, December 19, 2024 (Chairman: Prof. A.N. Svistunov). All patients provided written informed consent prior to participation.

The study is a prospective, single-center, randomized trial that included 29 patients with postoperative stump wound suppuration following lower limb amputation and patients with diffuse phlegmon of the lower extremities who received treatment in the purulent-surgical unit at the A.K. Eramishantsev City Clinical Hospital from December 2024 to June 2025. The inclusion criteria for the study were age over 18 years and symptoms of a purulent-inflammatory soft tissue disease of the trunk or extremities. Exclusion criteria were stage II-III dyscirculatory encephalopathy, decompensated cardiovascular and respiratory diseases, coagulopathy, thrombocytopenia, psychiatric disorders, and patient refusal.

**Figure 1:**
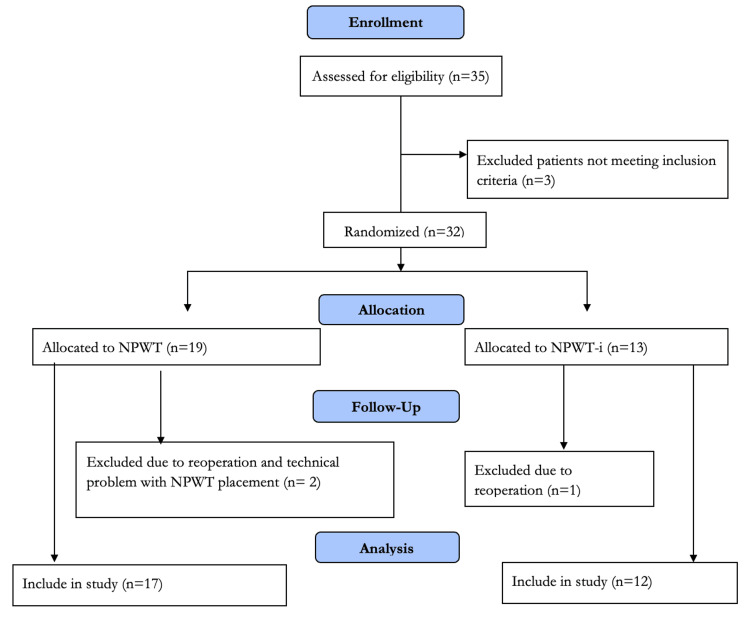
Consort flow diagram showing selection of the study's participants Of the 32 randomized patients, 2 (1 from each group) were excluded due to unplanned re-operations for vital indications, and 1 patient was excluded due to persistent technical failure to maintain an airtight NPWT seal. Consequently, 29 patients (17 in the NPWT group and 12 in the NPWT-i group) were included in the final per-protocol analysis.

Of 35 patients assessed for eligibility, 3 were excluded for not meeting the inclusion criteria (specifically, refusal to provide informed consent). The remaining 32 patients were randomized into two treatment groups using the envelope method. Group I (n=17) received NPWT as part of their comprehensive treatment, while Group II (n=12) received NPWT-i. During the follow-up period, three patients (9.4%) were discontinued from the study. Two discontinuations resulted from reoperation (one in each group), and one was due to technical failure of the NPWT device (inability to maintain airtightness). Consequently, the final analysis included 17 patients in the conventional NPWT group and 12 in the NPWT-i group. Planimetric analysis of serial wound photographs was performed by an independent researcher who was blinded to the patient's treatment group allocation. The complete participant flow is detailed in Figure 1.

Baseline characteristics

Baseline demographic and clinical characteristics of the two groups are summarized in Table 1. All p-values exceeded 0.05, indicating no statistically significant differences between Group I (NPWT) and Group II (NPWT-i) in baseline characteristics. Fisher's exact test confirmed comparable distributions in sex (p=0.876), diabetes mellitus prevalence (p=0.774), microbial spectrum (*Staphylococcus (S.)* *aureus)* p=1.000, *Pseudomonas (Ps.) aeruginosa *p=1.000, *Klebsiella (Kl.) pneumoniae *p=0.628, *Acinetobacter (A.) baumannii* p=1.000), and treatment indications (p=0.723 for both categories). These results confirm successful randomization and baseline homogeneity between groups.

**Table 1:** Baseline characteristics of the study groups Statistics analysis performed with Microsoft Office Excel 2013 (Microsoft Corporation, Redmond, WA, US) and SPSS Statistics Ver. 20 (IBM Corp., Armonk, NY, US) software.

Sign	Group I (n=17)	Group II (n=12)	Total	p-value
Age, years	56.7 ± 24.3	63.3 ± 8.2	60.2 ± 16.25	0.313
Men, n	9	6	15	0.876
Women, %	8	6	14	0.876
Wound size, cm^2^	94.7± 20.1	88.9 ± 27.3	92.8 ± 31.6	0.539
Diabetes mellitus, n	9	7	16	0.774
Type of pathogen, n				
S. aureus	7	5	12	1.000
Ps. aeruginosa	5	3	8	1.000
Kl. pneumonia	2	2	4	0.628
A. baumannii	3	2	5	1.000
*Indications for vacuum system use, n*				
Suppuration of the postoperative wound in a thigh or lower leg stump	9	5	14	0.723
Phlegmon of the left lower extremity	8	7	15	0.723

The obtained numerical data underwent statistical processing using methods of medical statistics in accordance with modern requirements. Continuous variables are presented as mean ± standard deviation (M ± SD) if normally distributed, or as median and interquartile range (Me (Q25; Q75)) if non-normally distributed. Normality was assessed using the Shapiro-Wilk test. Intergroup comparisons for normally distributed data were performed using Student's t-test, while the Mann-Whitney U test was used for non-normally distributed data. Categorical variables are presented as counts (percentages) and were compared using Fisher's exact test. A two-sided p-value of <0.05 was considered statistically significant. Computations were performed using Microsoft Office Excel 2013 (Microsoft Corporation, Redmond, WA, US) and SPSS Statistics Ver. 20 (IBM Corp., Armonk, NY, US) software.

Negative pressure wound therapy techniques

**Figure 2:**
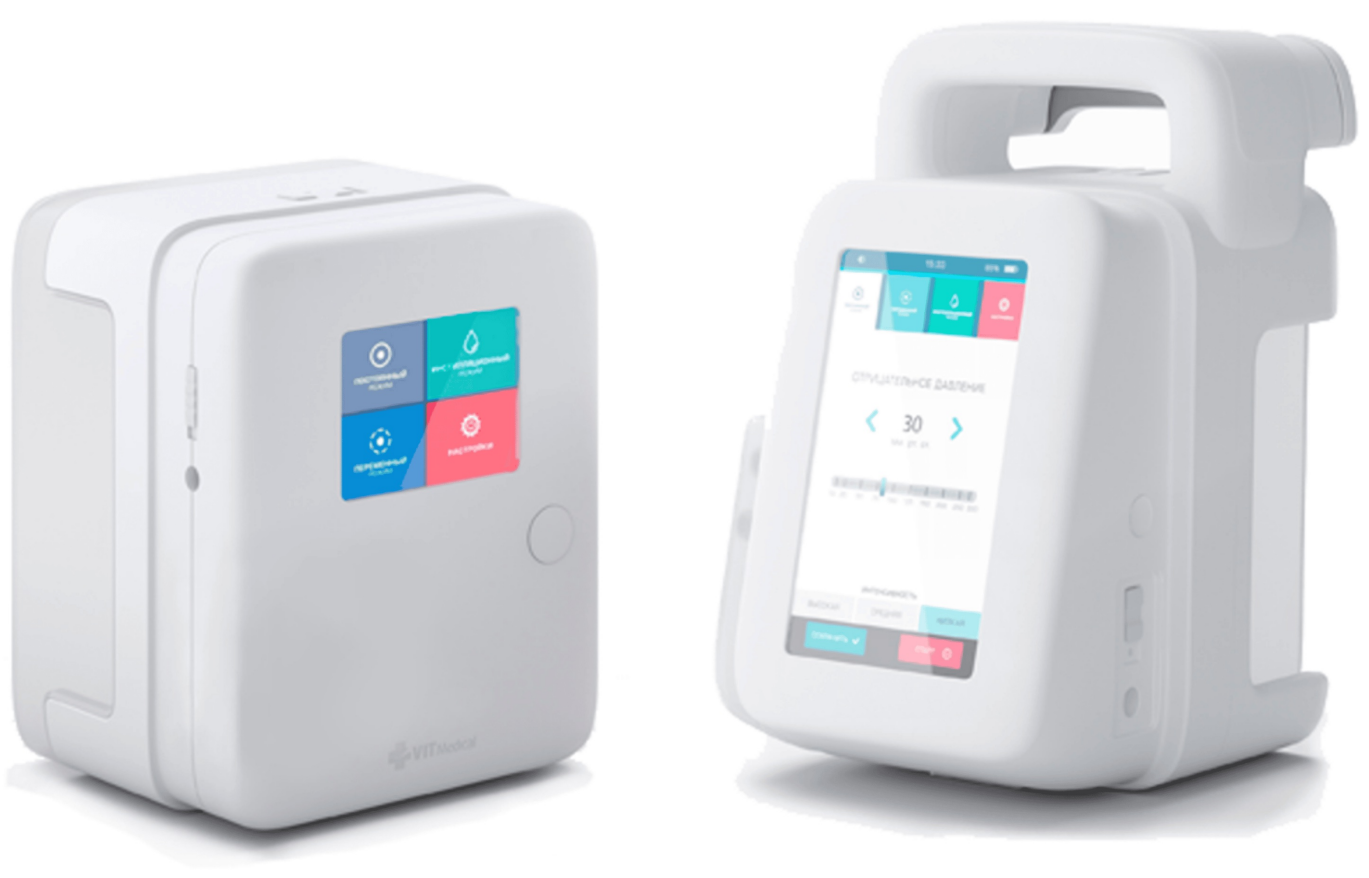
VIT devices for negative pressure wound therapy A. VIT Mobil device: has a canister option of 400 or 700 ml, provides a programmable negative pressure range from 10 to 300 mmHg, and supports two operating modes - continuous and intermittent. B. VIT Ultra device has a canister capacity of 2000 ml, provides a negative pressure range from 10 to 300 mmHg, and has three operating modes - continuous, intermittent, and instillation.

For patients in Group I, the "VIT Mobil" device (VIT Medical, Moscow, Russia) was used, set to a continuous mode of operation with a negative pressure of 125 mmHg. In Group II, the "VIT Ultra" device (VIT Medical) was applied; its use allows for the convenient and reliable coordination of the phases of vacuum-instillation therapy due to the programmability of parameters such as the level and duration of negative pressure, volume and rate of solution administration, as well as dwell time (Figure 2).

Vacuum-instillation therapy in patients of group II

The NPWT-i cycle parameters (duration, volume, dwell time) were configured per the manufacturer's (VIT Medical) guidelines for the 'VIT Ultra' system and reflect the standardized institutional protocol for instillation therapy. Each device cycle (A) lasted 2 hours and 40 minutes and included three phases: application of negative pressure at −125 mmHg for 2 hours and 30 minutes, instillation of 15 ml of normal saline for 20 seconds (as recommended by the manufacturer), and a dwell period of 10 minutes during which the solution remained in the wound. Patients received three such NPWT-i treatment cycles (B) per day. The vacuum dressing was changed every three to four days (C), always ensuring that at least three consecutive days of instillation therapy were completed before each change. At the time of dressing replacement on days 3-4, planimetric wound analysis was performed (D). After this stage, therapy was continued in the form of negative pressure without instillation (E) [11]. The sequence of interventions is outlined in Table 2.

**Table 2:** Protocol for applying the method of negative pressure wound therapy with instillation (NPWT-i) of normal saline for the entire treatment period Phase A: Included three phases - application of negative pressure at −125 mmHg for 2 hours and 30 minutes, instillation of 15 ml of normal saline for 20 seconds, and a dwell period of 10 minutes during which the solution remained in the wound; Phase B: Three NPWT-i treatment cycles per day; Phase C: Vacuum dressing replacement; Phase D: Planimetric wound analysis

Days of the week for treatment	Treatment phases for each day							
Monday	A	B	-		D	B	-	
Tuesday	C	B	-		C	B	-	
Wednesday	C	B	-		C	B	-	
Thursday	-		D	B	-		D	B
Friday	-		C	B	-		C	B
Saturday	-		C	B	-		C	B
Sunday	-		C	E	-		C	E

Postoperative treatment for all patients included systemic antibiotic therapy selected based on the diagnosis, suspected pathogen, and subsequently adjusted according to the results of bacteriological analysis of the exudate. Patient management involved a multidisciplinary team comprising, in addition to a surgeon, a therapist, an endocrinologist, and a clinical pharmacologist.

**Figure 3:**
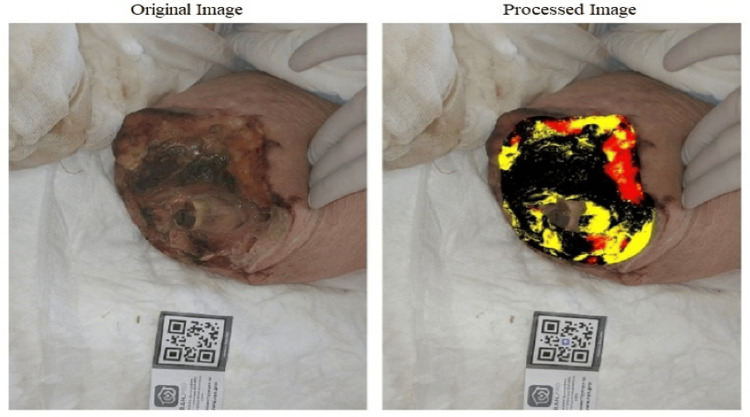
Planimetric analysis and calculation of the wound area in the program "RAN.PRO " (Russia) Distribution by tissue type: Fibrin (27%), Granulation (12%), Necrosis (61%) Areas: Total (95.1 cm²), Fibrin (25.5 cm²), Granulation (11.7 cm²)

To assess the wound-healing process, planimetric analysis of the wound surface was performed using a smartphone and the mobile application "RAN.PRO" (Russia) (Figure 3). This software allows for an objective evaluation of the effectiveness of the wound management methods employed. The algorithm differentiates tissue types based on chromatic and textural analysis: necrotic tissue (dark/black areas), fibrinous exudate (yellow/gray areas), and viable granulation tissue (red/pink areas). The application rapidly calculates the dimensions of the wound's structural elements: the total area of necrotic tissue, fibrin, and granulation tissue, expressed in both absolute and relative values [12].

To evaluate the effectiveness of the wound treatment methods used, on day 7 after the initial surgical debridement, we determined the serum level of C-reactive protein (CRP) [13]. In both groups, the number of secondary surgical debridements and the duration of patient hospitalization were also analyzed.

## Results

**Figure 4:**
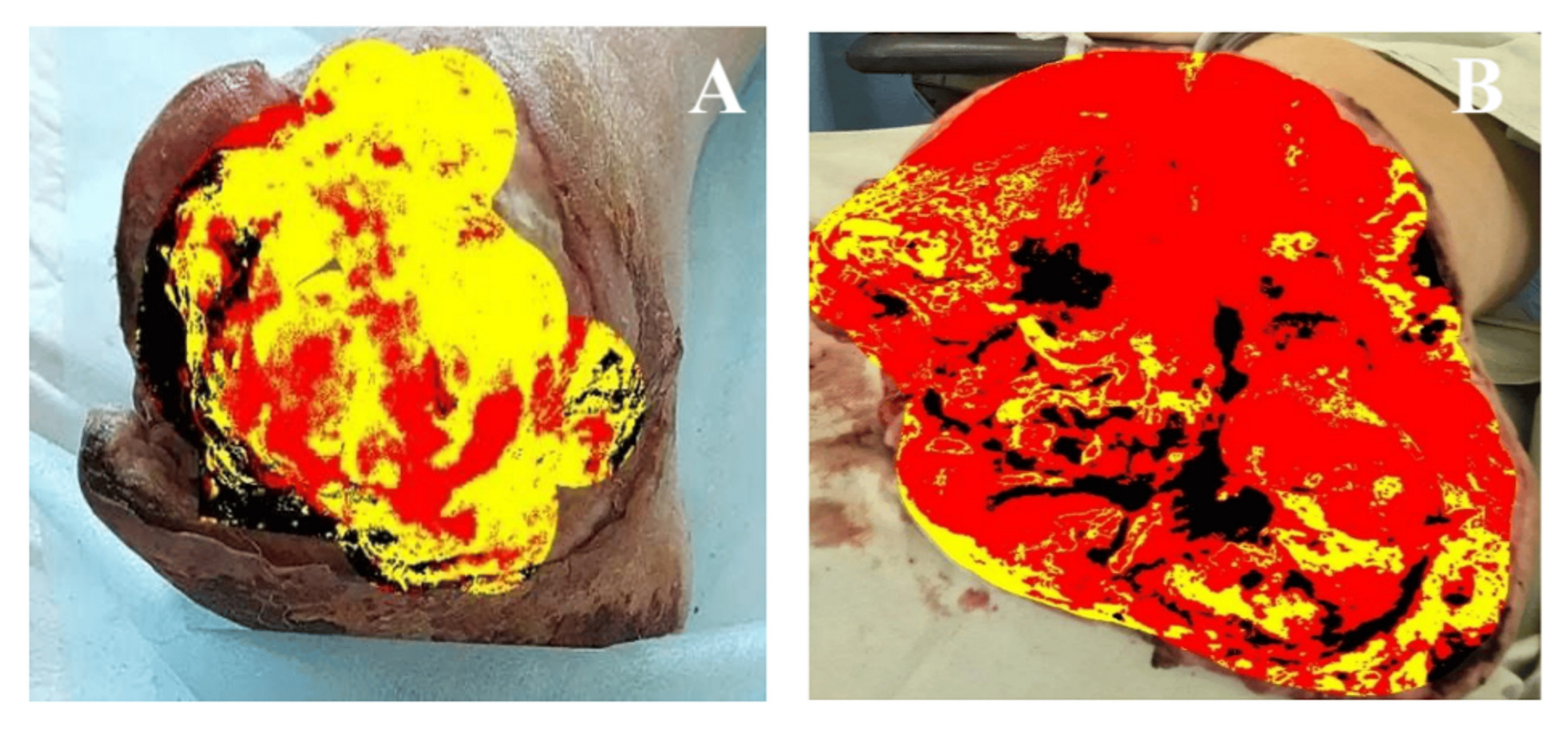
Wound changes on day 7 after primary surgical treatment in patients of both groups A. Group I; B. Group II

The appearance of wounds in patients from both groups on day 7 after initial surgical debridement is shown in Figure 4.

**Figure 5:**
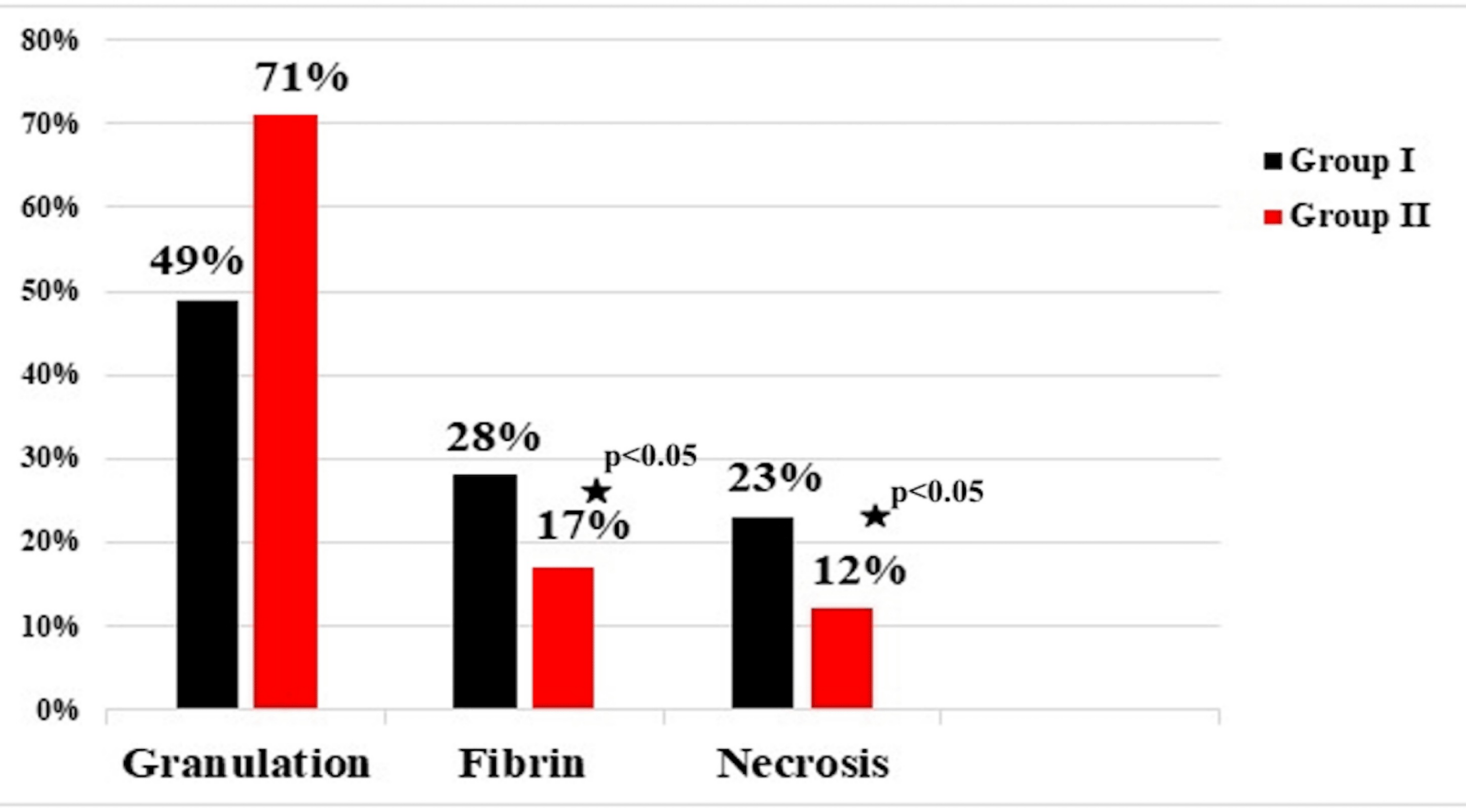
Results of planimetric analysis of wounds on day 7 after primary surgical debridement (* - when p<0.05)

As can be seen in Figure 4, by day 7 after initial surgical debridement, patients from Group II exhibited a larger area of granulation tissue and a smaller wound area covered by fibrin. The results of the planimetric analysis of the wound surface on day 7 after initial surgical debridement for patients in both groups are presented in Figure 5.

As shown in Figure 5, during these periods, the area of fibrin and necrosis in the wound was statistically significantly smaller in Group II patients who were treated with the NPWT-i method. Furthermore, the amount of granulation tissue in this group's patients was significantly greater. This indicates the efficacy of NPWT-i.

The mean plasma C-reactive protein level on day 7 after surgical debridement was significantly higher in Group I than in Group II (96.3 ± 14.2 mg/L vs. 49.4 ± 29.1 mg/L, respectively; p < 0.05). The mean number of secondary surgical wound debridements was 5.4 ± 1.2 in Group I and 3.1 ± 0.7 in Group II (p < 0.05). As can be seen, the number of secondary surgical debridements was significantly lower in Group II patients (NPWT-i). The mean duration of hospitalization was 41.7 ± 6.1 days in Group I and 32.5 ± 4.2 days in Group II (p < 0.05).

## Discussion

NPWT promotes wound healing by reducing lateral tissue tension (by approximately 50%) in a sutured wound, increasing blood flow, and reducing tissue edema [14-17].

The meta-analysis by Grant-Freemantle et al., which included 9 studies (1095 patients) published before 2019 with data on the primary outcome of deep infection, reported 55 cases of infection among 614 patients in the NPWT group and 84 infections among 481 patients in the conventional dressing group. It was shown that the use of NPWT in the treatment of open fractures reduces the likelihood of deep infection and split-thickness graft loss compared to conventional dressings [18].

In our study, over 42% of patients in each group underwent lower limb amputation with subsequent postoperative wound suppuration. According to the literature, the readmission rate following major lower limb amputations ranges from 18% to 29%, while the surgical site infection rate varies from 13% to 28.6%. The mortality rate for major lower limb amputations is high, reaching 8.8% within 30 days after surgery and up to 47.9% within 1 year [18,19].

Vaddavalli VV et al. report a significant reduction in recovery time, an acceleration of prosthesis fitting timing, and a higher mental health score in the patient group treated with NPWT. This can be explained by reduced discomfort or pain due to fewer dressing changes. This method helps reduce wound-related complications and shortens rehabilitation time [20,21].

A 2023 meta-analysis by De Pellegrin et al. demonstrates that NPWT-i outperforms standard NPWT or conventional dressings in wound treatment regarding parameters such as the rate of complete wound healing and the reduction of complications. However, the limited quality of the analyzed studies indicates a need for future randomized trials to confirm these benefits and to identify the most suitable NPWT-i operating regimens in surgery [8].

Conversely, a 2023 meta-analysis by Tarricone A. et al. found no statistically significant differences in outcomes between NPWT-i and standard NPWT groups in the treatment of diabetic foot ulcers [22]. The authors suggest two explanations for this. First, thorough initial surgical debridement is such a critical factor that its effectiveness may overshadow the potential additional benefit of instillation. Second, the instillation protocol itself (a low dose of antiseptic or insufficient exposure time) may have been inadequate to achieve a measurable clinical effect. In our opinion, the success of diabetic foot syndrome treatment depends on its specific form.

The results obtained in our study demonstrate the advantages of NPWT-i over standard NPWT therapy in treating purulent-inflammatory soft tissue diseases. The statistically significant reduction in the number of repeated surgical debridements, the faster decrease in CRP levels, and the accelerated formation of granulation tissue in the wounds indicate more effective wound cleansing and stimulation of reparative processes when using NPWT-i.

Our findings are supported by data from other studies demonstrating the efficacy of instillation vacuum therapy in reducing wound bacterial burden and optimizing conditions for tissue regeneration [7-9].

The reduced length of hospitalization further confirms the practical and economic efficiency of this method. However, further multicenter studies are required to determine the optimal instillation parameters (type of solution, duration of exposure) and to assess long-term outcomes (recurrence rate, functional recovery).

The main limitations of the present study are the relatively small number of cases and its conduct at a single medical center. There is a lack of surgeon and patient blinding (performance bias) inherent to the physical nature of the interventions. The unequal final group sizes (17 vs. 12), resulting from post-randomization exclusions, may affect the power for analyzing some secondary endpoints. Also, the short-term follow-up period focused on early healing phases, which precludes conclusions regarding long-term outcomes such as recurrence rates, scar quality, or functional recovery. The use of normal saline as the sole instillation agent limits generalizability to other solutions (e.g., antiseptics, surfactants, or enzymatic solutions). Despite this, the presented data provide compelling evidence for the high clinical value of the NPWT-i method using normal saline in the treatment of severe purulent-inflammatory soft tissue diseases and justify its implementation in clinical practice.

## Conclusions

Negative pressure wound therapy with instillation of normal saline, compared to vacuum therapy without instillation, has been shown to be more effective in the comprehensive treatment of patients with purulent-inflammatory diseases of the soft tissues of the trunk and extremities. This is demonstrated by its ability to reduce the number of repeated surgical debridements, lead to a rapid decrease in serum C-reactive protein levels, reduce the area of necrotic tissue and fibrin in the wound, accelerate the formation of granulation tissue, and shorten the duration of patient hospitalization. This justifies its consideration as an advanced therapeutic option, particularly for the management of extensive purulent wounds where efficient and early wound-bed preparation is critical. Further large-scale, multicenter trials are warranted to confirm these preliminary findings, evaluate long-term outcomes, and establish standardized instillation protocols.
